# How the First Medical Imaging Cancer Atlas EUCAIM Was Populated: The Experience of a Reference Hospital.

**DOI:** 10.12688/openreseurope.21016.2

**Published:** 2025-11-24

**Authors:** Ana Penadés Blasco, Leonor Cerdá Alberich, Ana de Marco García, Carina Soler Pons, Irene Marín Radoszynski, Ricard Martínez, Damián Segrelles-Quilis, Ignacio Blanquer, Luis Martí-Bonmatí

**Affiliations:** 1 Biomedical Imaging Research Group (GIBI230), La Fe Health Research Institute, Valencia, Spain, 46026, Spain; 2 IRTIC- Instituto Universitario de Investigación de Robótica y Tecnologías de la Información y Comunicación, Valencia, Spain, 46980, Spain; 3 Instituto de Instrumentación para la Imagen Molecular, Universitat Politècnica de València, Valencia, Spain, 46011, Spain; 4 Medical Imaging Department, La Fe University and Polytechnic Hospital, Valencia, Spain, 46026, Spain

**Keywords:** federated infrastructures, sustainability, medical imaging, Artificial Intelligence, cancer research, data governance, innovation

## Abstract

The fragmentation and decentralization of medical data, including radiological imaging, continue to challenge large-scale observational research across Europe. Artificial Intelligence (AI) applied to big datasets is transforming diagnosis and treatments towards precision medicine across many diseases, yet the lack of findable, accessible, and interoperable datasets still limits model development, validation, and final clinical translation. The European Federation for Cancer Images (EUCAIM) project was launched in 2023 to address these challenges by establishing a secure centralized and federated infrastructure for the secondary use of large-scale oncological imaging and related clinical data.

By consolidating fragmented datasets, EUCAIM lays the groundwork for harmonized data governance and trusted cross-border sharing. Implementing a robust documentation framework is essential to ensure regulatory compliance, safeguard data integrity, and support secure data flows across institutional and national boundaries, fully aligned with European regulations and ethical standards.

EUCAIM builds on the AI for Health Imaging (AI4HI) initiative (Predictive In-silico Multiscale Analytics to support cancer personalized diagnosis and prognosis, empowered by imaging biomarkers - PRIMAGE, Accelerating the lab to market transition of AI tools for cancer management - CHAIMELEON, Novel pan-European imaging platform for artificial intelligence advances in oncology - EuCanImage, An AI Platform integrating imaging data and models, supporting precision care through prostate cancer’s continuum - ProCancer-I, A multimodal AI-based toolbox and an interoperable health imaging repository for the empowerment of imaging analysis related to the diagnosis, prediction and follow-up of cancer - INCISIVE and integrates over 94 partners and more than 180 stakeholders spanning medical imaging, high performance computing, data standardization, innovation, and legal compliance. This large collaborative ecosystem reinforces EUCAIM’s role as a reference for General Data Protection Regulation (GDPR) and European Health Data Space Regulation (EHDSR) adherence.

This publication presents the real-world experience of integrating imaging and clinical data from a reference university hospital into the EUCAIM infrastructure. It outlines the procedural, ethical, and legal challenges encountered, and details the strategies implemented to ensure compliance with data protection regulations, including privacy, security, and ethical standards. These insights offer a practical framework for future large-scale oncological imaging datasets harmonization and AI development, contributing to scalable, reproducible, and legally compliant research that strengthens Europe’s capacity for trustworthy AI-driven oncology solutions.

## Plain Language Summary

The EUCAIM (European Federation for Cancer Images) project was launched in 2023 to improve the way cancer imaging and related clinical data are shared and reused across Europe. Medical data, such as radiology images, are often fragmented and stored in different places, making it hard for researchers to access large, high-quality datasets. This limits the development, testing and validation of artificial intelligence (AI) tools that could help improve diagnosis and treatment.

EUCAIM aims to solve these difficulties by creating a secure and centralized infrastructure that brings together cancer imaging data from across Europe. It supports the safe and ethical reuse of these data for research, following strict rules on privacy, security, and informed consent. The project includes over 94 partners and 180 stakeholders from different fields (such as medical imaging, computing, data standards, and law) ensuring strong collaboration and extensive expertise.

EUCAIM builds on previous European projects (like PRIMAGE, CHAIMELEON, and ProCancer-I) and aligns with major EU regulations like the General Data Protection Regulation (GDPR) and the European Health Data Space Regulation (EHDSR). A key part of the project is ensuring that the infrastructure meets all legal and ethical requirements for handling sensitive health data.

This article shares the real-life experience of a reference hospital contributing imaging and clinical data to EUCAIM and describes the technical and legal steps taken to ensure compliance and protect patients' information. These insights offer a practical example for other institutions considering participation in similar initiatives.

Overall, EUCAIM is helping to build a trusted European framework for using medical imaging data in AI research, aiming to accelerate innovation in cancer care while protecting patients’ rights.

## Introduction

Access to secondary use of medical imaging data for AI-driven research are hindered by fragmentation, lack of interoperability, and complex regulations across Europe regarding privacy and security issues. Although many research initiatives have created imaging repositories, these are often project-specific, temporally limited, and constrained in their potential for reuse. This is especially challenging in oncology ^1– 3^, where AI tools for precise diagnosis, prognosis and treatment estimations require large, standardized and annotated datasets to reach full clinical applicability ^4, 5^.

The European Federation for Cancer Images (EUCAIM) project addresses these challenges by developing a large-scale, secure, and federated digital infrastructure for medical imaging and related clinical data. EUCAIM accelerates AI research in personalized medicine, offering tools that support clinical decision-making in complex settings ^6^. EUCAIM builds on the AI for Health Imaging (AI4HI) network, which includes five major Horizon Europe projects (PRIMAGE, CHAIMELEON, EuCanImage, ProCancer-I, and INCISIVE). With over 90 partners and more than 180 stakeholders, EUCAIM is creating a GDPR-compliant, sustainable ecosystem for AI-based cancer research ^7, 8^.

While EUCAIM offers a unique opportunity to harmonize and share annotated oncological imaging data, its success depends on overcoming technical barriers (standardization, transformation, anonymization, curation), strict compliance with the General Data Protection Regulation ( GDPR), the European Health Data Space Regulation ( EHDSR), and the Artificial Intelligence Act (AI Act), and with a clear governance frameworks ^9^. The EUCAIM platform aims to host over 60 million images from more than 100,000 patients, transforming medical data use in personalized actionable care ^10^.

EUCAIM’s hybrid model allows Data Providers to transfer data to a reference node or set up their own federated node. This paper analyses the use case of real-world data transfer from a referral hospital, detailing workflows and practical solutions to secure legal compliant integration into EU-wide repositories.

## Material and methods

Data integration in EUCAIM follows either a *Data Sharing Agreement* (DSA), where data stays within the institution via a federated node; or a *Data Transfer Agreement* (DTA), where data is physically moved to a reference node which comprises 10 Gigabyte R283-ZF0-AAL1 nodes, each equipped with two AMD EPYC 9474F 48-core processors (a total of 960 cores), 7.68 TB of RAM, 15 NVIDIA A30 and 10 NVIDIA L40S GPU accelerators with 48 GB of RAM each, as well as an additional storage server with 16 TB of NVMe SSD disks connected to the nodes via dual 25 GbE links. In the experiments performed in previous works ^11^, this capacity is sufficient to deal with a workload of over 30 concurrent users.

For the retrospective imaging data included in this study, no informed consent was required, an exemption of informed consent was submitted to and approved by the Ethics Committee. All these nodes are core to EUCAIM’s infrastructure.

### Data extraction and exposure pipeline

To ensure standardized, anonymized, and compliant data integration into the EUCAIM infrastructure, a structured Extraction, Transformation, and Loading (ETL) pipeline was defined, incorporating best practices in data governance, security, and regulatory adherence. Imaging data were extracted from the hospital Picture Archiving and Communication System (PACS) through specific nodes in Digital Imaging and Communication in Medicine (DICOM) format according to each project’s inclusion criteria. Corresponding clinical data were retrieved from the Electronic Health Record (EHR) system to maintain proper alignment. Pseudonymization and compliance checks were carried out on a restricted-access virtual machine by dedicated personnel, ensuring a clear separation from researchers and full GDPR compliance. This process follows guidelines from the DICOM Standards Committee and the AI4HI initiative to guarantee interoperability with EUCAIM.

Key pseudonymization steps included replacing the PatientID with the project code (for both imaging and clinical data) and applying the Blake2b hashing function to replace the AccessionNumber and StudyID metadata. Folder structures were adjusted to remove any Personally Identifiable Information (PII), and all public/private DICOM metadata potentially containing personally identifiable information were removed, following EUCAIM’s anonymization profile. Secondary captures and screenshots, which may embed sensitive information, were excluded when identified by terms like “DERIVED,” “SECONDARY,” or “SCREEN SAVE” in the ImageType metadata.

**Figure 1.**
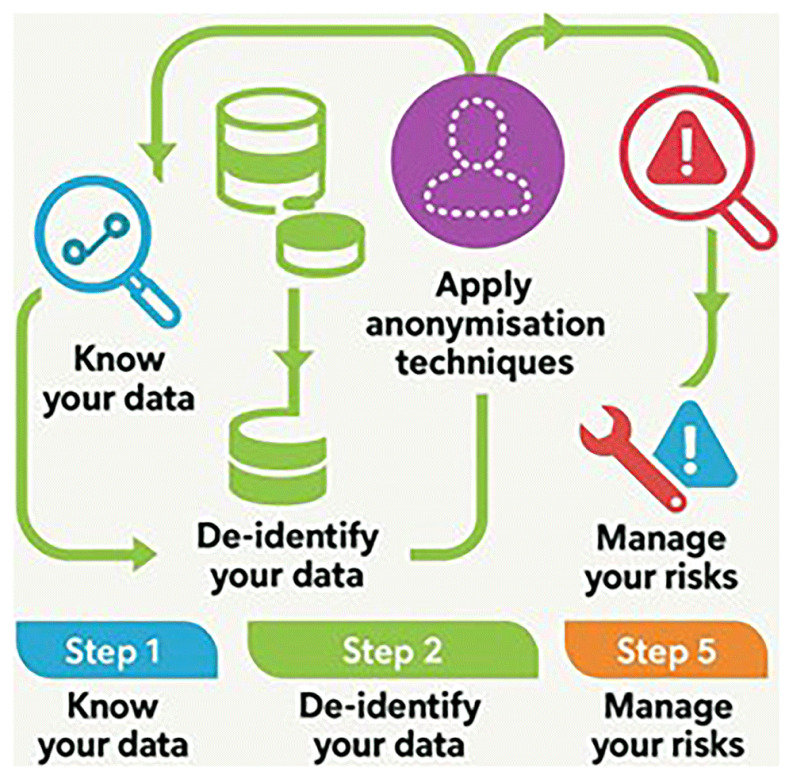
Steps for anonymization. Source: Adapted from AEPD. Guide to basic anonymization. Prepared by the National Data Protection Authority of Singapore (PDPC - Personal Data Protection Commission Singapore.

Pseudonymized data was then transferred to a separate file system, the Pseudonymized Medical Imaging Repository (RIMP). The DICOM File Integrity Checker developed by our group was used to detect corrupted or missing files. Data were finally ingested into EUCAIM datasets through the QP-Insights API, ensuring stronger anonymization, and secure transfer or sharing. This two-layer anonymization approach mitigates re-identification risks, aligning with procedures recommended by the Spanish Data Protection Agency and best practices from the Singaporean authority ( Figure 1). For data standardization, all DICOM files and metadata were validated for compliance with EUCAIM’s structure and interoperability standards.

The EUCAIM Federated Node provides an open-source, fully integrated Data Lake, Registry, and Secure Virtual Research Environment, backed by dedicated computing resources. Its API enables the ingestion while logging all transfers for traceability and auditability. Federated nodes are fully integrated within the EUCAIM platform and meet the EHDSR requirements for Secure Processing Environments (SPEs). SPEs ensure that data processing stays local, under the health data holder’s continuous control, significantly reducing transfer risks. Specialized mediation software enforces strict anonymization rules, restricts user actions, and logs all activities to prevent misuse and ensure full accountability.

By leveraging SPEs and federated nodes, EUCAIM provides a robust, compliant, and auditable framework for the secure secondary use of health data. This rigorous ETL pipeline ensures scalability, data quality, and legal compliance, positioning EUCAIM’s reference and federated nodes as the backbone for integrating data from research projects, clinical trials ^12^, and future initiatives, in line with European regulations such as the Data Act and Data Governance Act. This strategy guarantees EUCAIM’s long-term sustainability and impact on AI research in medicine ^13^.

### Documentation framework for data integration to EUCAIM

Ensuring compliant data integration within EUCAIM requires strict adherence to clear documentation protocols. At its current stage, decisions on the final integration of a dataset and/or federated node are verified by an Access Committee (AC). During this phase, it is mandatory to provide documentary evidence that guarantees accountability in EUCAIM’s operation and demonstrates the application reliability.

For data transfers to EUCAIM, the following documents are considered to safeguard data integrity and regulatory compliance previous to the signature of the DTA:

- Data Protection Officer (DPO) Report or Self-Declaration: formal declaration by the transferring institution affirming that the data processing activities comply with GDPR requirements, including data minimization, data protection by design, and risk mitigation measures.
- Codes of conduct or certification schemes adherence: a non-mandatory certification of GDPR declaring the institution provides evidence of adherence to established codes of conduct or certifications relevant to GDPR compliance.
- Data Protection Impact Assessment (DPIA). documented evidence of the completed DPIA must be submitted in cases where, in accordance with Article 25 of the GDPR and the relevant national authority's whitelist, it is required.
- Ethical Approval Documentation from the relevant Ethics Committee: certifying that the data transfer aligns with ethical research practices and data protection regulations. The requirement for ethics approval, its specific content, or any granted exemption is determined by national law. In cases where ethics approval is not required, it is recommended to be explicitly documented in the statement provided by the DPO.
- Legal Representation Declaration: signed by the institution’s legal representative, confirming that the data transfer adheres to legal obligations and that the representative is duly authorized to sign agreements on behalf of the institution. This documentary requirement is crucial, as requests might be handled by researchers or individuals who lack the legal capacity to make binding declarations of intent.

For Data Sharing Agreements (DSAs) via federated nodes, the same requirements apply, with an additional Security Report or certification (e.g., ISO 27001) document verifying that data sharing meets EUCAIM’s security standards for encryption, user authentication, and access control.

All documentation must be verifiable and may undergo audits. Federated nodes must show secure operations and technical interoperability with the EUCAIM platform. Likewise, datasets claimed to be anonymized will be checked: any re-identification risk means the data will be returned for further processing until fully compliant. This framework guarantees that all partners contribute data responsibly, supporting secure, transparent, and efficient data sharing within the EHDSR.

**Figure 2.**
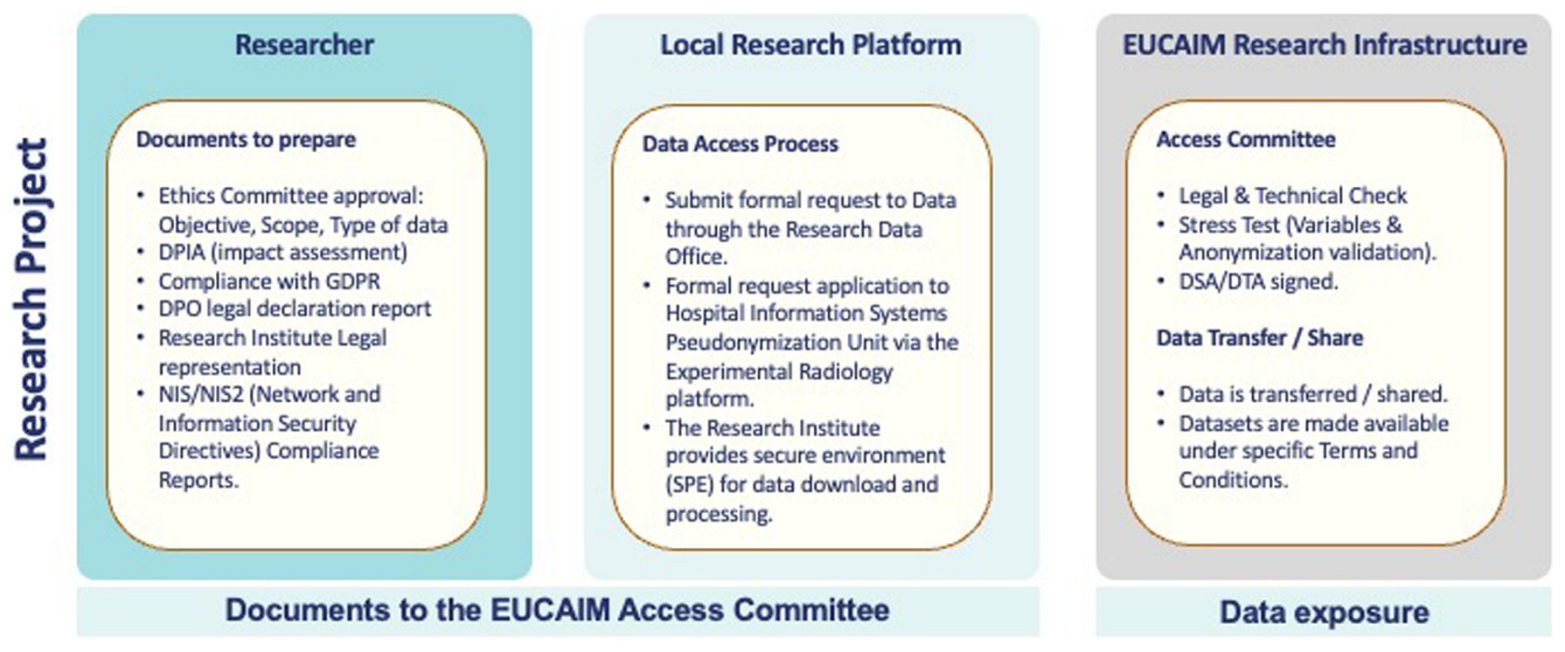
Summary of the relevant legal documentation in EUCAIM.

The relevant legal documentation can be summarized in Figure 2

### Use case of data transfer from completed research projects

The PRIMAGE project (GA: 826494) demonstrated how historical datasets could be successfully transferred and validated within the EUCAIM reference node. The main steps included:

- Legal and Ethical Compliance: all approvals and documentation were secured.
- Data Processing & Transfer: imaging data were extracted, anonymized, and reformatted into EUCAIM-compliant DICOM structures. Clinical data was similarly processed, aligned with the EUCAIM Construction, Design and Management (CDM) and hyper-ontology based on mCODE specifications.

### Use case of data transfer from oncology clinical trials

The previous approach was also applied to ongoing clinical trials, expanding EUCAIM’s reference node cases. Additional challenges emerged, particularly regarding data ownership and sponsor agreements. Key strategies included:

- Data Ownership Clarification: a legal framework defined that imaging data belong to the patient’s EHR, not the trial sponsor, minimizing transfer conflicts and ensuring EU compliance.
- Informed Consent: exemptions were requested as data were fully anonymized and restricted to research use.
- Periodic Scheduling: semi-annual transfers allow continuous integration in line with regulatory approvals.

### Use case of data sharing from ongoing research projects

The CHAIMELEON project (GA: 952172) illustrated a continuous dataset sharing process without disrupting ongoing research, serving as a model for federated nodes. For these projects, a more dynamic workflow was required due to evolving data collection and regulatory requirements:

- Ethics Committee Approval: an addendum updated the original approval to permit secondary data use in EUCAIM.
- Data Harmonization & Monitoring: a real-time tracking system ensured seamless, compliant integration.

CHAIMELEON reached a high maturity level by preparing a robust DSA, anonymizing data, implementing a secure node, and completing a DPIA, setting a strong precedent for future compliant transfers.

### Ethical and legal considerations

Each use case prioritizes full compliance with data protection regulations, especially GDPR. All data transferring and sharing activities were approved by the relevant ethics committees, ensuring confidentiality, integrity, and security. This rigorous approach has enabled the secure transfer and sharing of large volume of anonymized data, contributing to advanced AI research. Within EUCAIM, data use is strictly limited to users with approved research projects that meet institutional ethics standards and follow the Access Committee’s protocols.

The recent approval of the EHDSR marks a significant milestone for EUCAIM and similar initiatives, providing a harmonized framework for secure access, sharing, and secondary use of large-scale medical datasets. This framework sets clear rules for research and innovation, supported by strict requirements for security, privacy, and informed consent, fostering an interoperable and sustainable data ecosystem. EUCAIM’s activities have been progressively aligned with this evolving landscape, positioning as a pilot platform under HealthData@EU. Its formal integration into the EHDS ecosystem will strengthen data accessibility and protection, reinforcing the project’s long-term sustainability and scientific value, as data volume and diversity grow.

EUCAIM addresses sustainability through the establishment of a European Digital Infrastructure (EDIC) to enable multi-country investments in large-scale projects. The proposed model combines national and node contributions with European funding to ensure long-term operation of the Central Hub and integration of new partners.

## Results

The implementation of a dedicated, harmonized pipeline for data transfer and sharing enabled the successful ingestion of cancer imaging data into the EUCAIM infrastructure. This process established a replicable and scalable workflow suitable for future large-scale federated data integration.

In total, 12,484 medical imaging studies at our hospital and research institute were identified, reviewed, and prepared for integration into EUCAIM, covering both research datasets and real-world clinical trial data. Of these, 10,892 studies (87.24%) from 6,105 patients have been fully processed and validated for upload through either a reference or federated node approach. Our institution has contributed to several initiatives, including the completed PRIMAGE project with 878 studies, the ongoing CHAIMELEON project with 5,346 studies, and a series of Oncology Clinical Trials comprising 4,668 studies. The datasets include multiple modalities ((Computed Tomography - CT, Magnetic Resonance - MR, mammography, Positron Emission Tomography-Computed Tomography - PET-CT)) and represent a broad spectrum of cancer cases and patient populations ( Table 1).

**Table 1.** Overview of prepared Data Sources for Integration into the EUCAIM Reference Node and Federated Node.

Acronym	Project status	Number of identified imaging studies	Number of prepared imaging studies	Node
PRIMAGE	Completed	878	878	Reference
CHAIMELEON	Ongoing	5,346	5,346	Federated
Clinical Trials	Completed and ongoing	6,260	4,668	Reference

The ETL pipeline achieved a processing efficiency of 98.6%, with minimal data loss. Automated DICOM anonymization, combined with manual checks, ensured GDPR compliance while maintaining clinical relevance. PRIMAGE delivered one of EUCAIM’s largest structured paediatric oncology datasets, setting a benchmark for rare disease integration. CHAIMELEON validated a stepwise model for harmonized data sharing, and the inclusion of ongoing clinical trial data demonstrated the feasibility of incorporating prospective datasets into the federated infrastructure.

Several challenges emerged that required refinements to optimize efficiency, scalability, and compliance. A key technical challenge was achieving interoperability across diverse imaging formats and legacy PACS metadata, which often required extensive transformation and custom mapping. Variability in clinical data mappings also highlighted the need for more automated and standardized processes.

Administrative and legal aspects added complexity. Ethics approvals often needed significant revisions depending on the project type, and requests for informed consent exemptions required careful justification and additional documentation. These barriers underscored the lesson learned: the EHDS will require organisations to renew their ethical and legal governance models to ensure predictable, efficient data sharing, including data holders, research infrastructures, and users.

Scalability was initially hindered by reliance on manual anonymization checks, slowing processing and introducing inconsistencies. Introducing automated validation tools and batch workflows significantly improved efficiency, though manual review remains necessary for nuanced cases like secondary capture images.

These insights guided refinements to our protocols. Automated metadata validation minimized errors, while standardized ethics templates helped accelerate approval timelines. Experience gained across projects emphasized the importance of early alignment with EUCAIM requirements, ensuring technical, compliance, and governance aspects are clear from the outset.

Despite initial hurdles, this process strengthened our capacity to securely transfer oncological imaging data with the highest standards of security, privacy, and interoperability, providing a solid foundation for future data-sharing efforts within EUCAIM and similar large-scale initiatives.

## Discussion

EUCAIM is established as a landmark initiative in oncological imaging, offering Europe’s first large-scale hybrid infrastructure for the secondary use of imaging, clinical, and molecular data. By integrating datasets from completed research, ongoing projects, and clinical trials, EUCAIM ensures alignment with the EHDSR and GDPR, thereby supporting the full potential of artificial intelligence in advancing predictive and personalized cancer care. Nevertheless, significant challenges remain, particularly data fragmentation, legacy system integration, and cross-border regulatory complexity.

A key barrier to building a unified repository has been the variability in data formats, metadata, and institutional governance practices. EUCAIM addresses this challenge through structured documentation frameworks and standardized ETL pipelines, which enhance data governance and promote interoperability across systems. Even so, harmonizing legacy PACS data and oncological metadata will continue to demand refinements and shared best practices.

From the outset, ethical and legal considerations have been central. Multi-step anonymization, robust audit logs, and clear governance models ensure compliance and traceability. However, achieving consistent ethics approvals and seamless cross-border data sharing still requires effort. The EHDS framework will be instrumental in easing these processes, but only if health data holders, research infrastructures, and users renew and align their governance models.

The lessons learned from large-scale data integration reinforce the value of early legal and technical alignment. PRIMAGE, CHAIMELEON and ongoing clinical trials each illustrate how proactive regulatory planning and clear data management frameworks enable scalable, compliant data sharing.

Long-term sustainability will rest on strict adherence to Findable, Accessible, Interoperable and Reusable (FAIR) principles, supported by reference and federated nodes, FAIR Data APIs, and persistent identifiers that guarantee data findability and reusability. Automating compliance checks and deploying secure cloud-based environments will be essential to handle ever-growing data volumes, while standardized metadata schemas will further support seamless reuse.

Through the continuously refinement of its governance strategies and proactive alignment with the evolving regulatory landscape under the EHDS, EUCAIM is positioned to become a cornerstone for AI-driven oncology research. Its robust infrastructure not only accelerates the clinical translation of AI tools but also strengthens a culture of trustworthy, collaborative data sharing across Europe, reinforcing EUCAIM’s pivotal role in shaping the future of cancer imaging and precision medicine. Central to this effort is the essential contribution of data providers, whose engagement ensures the availability, diversity, and quality of datasets reinforcing EUCAIM in shaping the future of precision medicine.

## Ethical approval statement

This manuscript has been approved by the Research Ethics Committee on Medicinal Products of La Fe University and Polytechnic Hospital under registration number 2022-437-1. For the retrospective imaging data included in this study, no informed consent was required, an exemption of informed consent was submitted to and approved by the Ethics Committee.

## Data Availability

No data are associated to this manuscript.
