# Supplementary material for: How the First Medical Imaging Cancer Atlas EUCAIM Was Populated: The Experience of a Reference Hospital
Source: Open Res Eur. 2025 Dec 8;5:310. Originally published 2025 Oct 10. [Version 3] doi: 10.12688/openreseurope.21016.3 (PMC12640480; doi:10.12688/openreseurope.21016.3)
Supplement: Peer review history

# Reviewer report: Reviewer response for version 3

**DOI:** 10.21956/openreseurope.23947.r65769

**Published:** 2026-01-01

**Authors:** Ana M. Barragán Montero

**Affiliations:** 1 Molecular Imaging, Radiation and Oncology (MIRO) Laboratory, UCLouvain, UCLouvain, Belgium

The authors have answered all my comments, very good job! Congratulations on this great initiative.

Is the case presented with sufficient detail to be useful for teaching or other practitioners?

Partly

Is the work clearly and accurately presented and does it cite the current literature?

Partly

If applicable, is the statistical analysis and its interpretation appropriate?

Not applicable

Are all the source data underlying the results available to ensure full reproducibility?

Not applicable

Are the conclusions drawn adequately supported by the results?

Yes

Is the background of the case’s history and progression described in sufficient detail?

Yes

Reviewer Expertise:

Artificial intelligence, radiation therapy, medical imaging, cancer treatment

I confirm that I have read this submission and believe that I have an appropriate level of expertise to confirm that it is of an acceptable scientific standard.

# Reviewer report: Reviewer response for version 3

**DOI:** 10.21956/openreseurope.23947.r65770

**Published:** 2025-12-30

**Authors:** Neha Neha, Deepak Kumar Shukla

**Affiliations:** 1 Rutgers Business School, Rutgers University Newark Business School (Ringgold ID: 33869), Newark, Jersey, USA; 2 Kent State University, Kent, USA

Author has made all the suggested changes.

Is the case presented with sufficient detail to be useful for teaching or other practitioners?

Partly

Is the work clearly and accurately presented and does it cite the current literature?

Yes

If applicable, is the statistical analysis and its interpretation appropriate?

Not applicable

Are all the source data underlying the results available to ensure full reproducibility?

Partly

Are the conclusions drawn adequately supported by the results?

Yes

Is the background of the case’s history and progression described in sufficient detail?

Yes

Reviewer Expertise:

Artificial IntelligenceComputer VisionImage ProcessingDeep learningMedical Imaging

We confirm that we have read this submission and believe that we have an appropriate level of expertise to confirm that it is of an acceptable scientific standard.

# Reviewer report: Reviewer response for version 2

**DOI:** 10.21956/openreseurope.23735.r64762

**Published:** 2025-12-02

**Authors:** Neha Neha, Deepak Kumar Shukla

**Affiliations:** 1 Kent State University, Kent, USA; 2 Rutgers Business School, Rutgers University Newark Business School (Ringgold ID: 33869), Newark, Jersey, USA

Author has made all the suggested updates.

Is the case presented with sufficient detail to be useful for teaching or other practitioners?

Partly

Is the work clearly and accurately presented and does it cite the current literature?

Yes

If applicable, is the statistical analysis and its interpretation appropriate?

Not applicable

Are all the source data underlying the results available to ensure full reproducibility?

Partly

Are the conclusions drawn adequately supported by the results?

Yes

Is the background of the case’s history and progression described in sufficient detail?

Yes

Reviewer Expertise:

Artificial IntelligenceComputer VisionImage ProcessingDeep learningMedical Imaging

We confirm that we have read this submission and believe that we have an appropriate level of expertise to confirm that it is of an acceptable scientific standard.

# Reviewer report: Reviewer response for version 1

**DOI:** 10.21956/openreseurope.22734.r62149

**Published:** 2025-11-22

**Authors:** Ana M. Barragán Montero

**Affiliations:** 1 Molecular Imaging, Radiation and Oncology (MIRO) Laboratory, UCLouvain, UCLouvain, Belgium

**Review of the manuscript Open Research Europe 2025, 5:310 “How the first medical imaging cancer atlas EUCAIM was populated: the experience of a reference hospital”**

**The manuscript describes the EUCAIM ecosystem and the methodology to submit images, illustrated with the experience of a hospital in the consortium. The article is well written and clear, and it is suitable for publication in the journal. However, I have some comments that I believe can help to increase the quality of the manuscript and before final publication.**

**MAJOR COMMENTS **

1. **Improve the contextualisation and illustrate the state of the art. In the first paragraph, they describe the current ecosystem for data sharing and the limitations, highlighting the need for an initiative like EUCAIM. While the paragraph is well written, I am missing a few concrete examples and references that support the arguments given in the paragraph. For instance, the authors mention that “many research initiatives have created imaging repositories, but they are often project-specific, temporally limited, …” but no example is given. Please provide at least two or three examples. Also what is the potential of EUCAIM with respect to other existing initiatives for large-scale data collection like TCIA ( https://www.cancerimagingarchive.net/), DESIRE ( https://www.straaleterapi.dk/en/desire/about-desire/), GrandChallenges ( https://grand-challenge.org/challenges/), etc I think a paragraph discussing this and giving specific examples will help the reader to better see the potential and value of EUCAIM**

 

1. **Describe more in detail the system for hosting  and using the data. The authors distinguish between two options (materials and method, paragraph 1): 1) DSA, where the data stays in the system, or DTA, where the data goes to the federated node. Can you provide more technical information about this part? Or either reference to already published protocols/papers describing this part? Some questions here: Why these two options and not only DTA directly? For hospitals choosing 1), is it up to them to buy a server to host the data? How is the connection then to other hospitals willing to use the data through the DSA? Does EUCAIM provide a technical team to make all these installations in the local computers or the hospital should do it? How can other hospitals use the DSA data to train models if the data did not leave the hospital? For DTA data, can I download the data into my servers to perform research (e.g. trained AI models)?**
** I understand that the paper here focuses on the population of the atlas, but some clarification (a paragraph will be enough) here is important, so that the community understands how the data can be used later. Otherwise, it seems a very nice platform to store data but the article does not give any clue about the potential to use the data later. **

 

1. **Disclose or add reference for already available material. This is published in Open Research journal, so I guess that the goal is to make as open as possible the results presented here. There are many parts where you mention that the authors (or the EUCAIM team) has developed a lot of material, but no reference is given. For instance, in material and methods “For data standardization, all DICOM files and metadata were validated for compliance with EUCAIM structure”, “The DICOM File Integrity Checker developed by our group” → is this material published elsewhere or made open-source through a github/github or similar repository? The amount of work that has been developed here has an incredible value for the community and should be made public whenever possible. **

 

1. **Pseudonymisation versus Anonymisation. I have a question regarding this, since both are mentioned during the text, it is not clear to me if the data uploaded is pseudonymised, and there is a log file somewhere (in the hospital submitting the data) that keeps a link (e.g. PatientID) between the data submitted and the real patient identity, or the data is fully anonymised. If the data is totally anonymised and there is no log-file, how do we prevent errors in the future if the hospital submit the same patient (e.g. submission in 2025 for project X, new patient ID projectX_01 - submission in 2026 of same patient for project Y, new patient ID project Y_01). Also, besides removing DICOM tags, can you describe if any action on the images has been done or not? There are some people that advocate to remove facial information from head-and-neck scans, but this can affect the potential use of the data (e.g. in head-and-neck cancer patients). **

 

1. **Data quality and documentation. You do mention DICOM compliance, anonymisation, etc, but what about data quality and documentation? **
  1. **About data quality, is there any metadata or any quality check to measure the quality of the submitted data? I am thinking more on the annotations of the data, for instance, if the annotations have been done by experts and following consensus international guidelines, etc A concrete example would be the adherence to contouring guidelines in radiotherapy images. Data annotations not being compliant to these guidelines and used to train models might carry future problems. Also, we could think about data quality of the images, if a hospital submit images with very low resolution, artefacts, etc. A way to go would be to enable a community rating for each dataset …**
  2. **About data documentation. I am putting myself on the user side, and the first question that comes to my mind is: how do I find and decide which data I use for my project? Having data documentation is very important, like a Data Sheet (similar to what they have done in https://arxiv.org/abs/1803.09010, https://datanutrition.org/, …). Is there any similar documentation standard in EUCAIM? **
2. **In general some paragraphs feel too much “GPT-style”, sometimes repeating the same but with different words. Please review and try to make the text less redundant and more concise whenever possible. **

Is the case presented with sufficient detail to be useful for teaching or other practitioners?

Partly

Is the work clearly and accurately presented and does it cite the current literature?

Partly

If applicable, is the statistical analysis and its interpretation appropriate?

Not applicable

Are all the source data underlying the results available to ensure full reproducibility?

Not applicable

Are the conclusions drawn adequately supported by the results?

Yes

Is the background of the case’s history and progression described in sufficient detail?

Yes

Reviewer Expertise:

Artificial Intelligence for medical imaging, radiotherapy treatment planning

I confirm that I have read this submission and believe that I have an appropriate level of expertise to confirm that it is of an acceptable scientific standard, however I have significant reservations, as outlined above.

# Response

**Published:** 2025-12-03

**Authors:** Ana Penades Blasco

**Affiliations:** Biomedical Imaging Research Group, La Fe Health Research Institute, Valencia, Spain, Spain

**Response to the REVIEWER #3 COMMENTS**

 Thank you very much for your comments and feedback. We have worked on improving the manuscript with your suggestions and comments.

**Reviewer #3:**

**Reviewer 3.1**: Improve the contextualisation and illustrate the state of the art. In the first paragraph, they describe the current ecosystem for data sharing and the limitations, highlighting the need for an initiative like EUCAIM. While the paragraph is well written, I am missing a few concrete examples and references that support the arguments given in the paragraph. For instance, the authors mention that “many research initiatives have created imaging repositories, but they are often project-specific, temporally limited, …” but no example is given. Please provide at least two or three examples. Also what is the potential of EUCAIM with respect to other existing initiatives for large-scale data collection like TCIA ( https://www.cancerimagingarchive.net/), DESIRE ( https://www.straaleterapi.dk/en/desire/about-desire/), GrandChallenges ( https://grand-challenge.org/challenges/), etc I think a paragraph discussing this and giving specific examples will help the reader to better see the potential and value of EUCAIM

**Authors:** Following the comments of the reviewer we now incorporated several sentences at the Introduction: “Multiple initiatives have previously generated valuable imaging repositories, examples include some Horizon Europe projects providing temporal available datasets such as Predictive In-silico Multiscale Analytics to support cancer personalized diagnosis and prognosis, empowered by imaging biomarkers - PRIMAGE, Accelerating the lab to market transition of AI tools for cancer management - CHAIMELEON, Novel pan-European imaging platform for artificial intelligence advances in oncology - EuCanImage, An AI Platform integrating imaging data and models, supporting precision care through prostate cancer’s continuum - ProCancer-I, and A multimodal AI-based toolbox and an interoperable health imaging repository for the empowerment of imaging analysis related to the diagnosis, prediction and follow-up of cancer - INCISIVE. And also some existing repositories such as The Cancer Imaging Archive (TCIA), which provides curated datasets but largely centred on disease-specific collections; GrandChallenges, where datasets are created to support competitions and are rarely updated or expanded once each challenge is completed; and domain-focused initiatives such as DESIRE in radiotherapy, which target specific clinical use cases and remain confined to their original scope. These projects have demonstrated the scientific value of shared imaging data, but they lack the harmonised governance, long-term sustainability mechanisms, and structured clinical context that are required for large-scale, reproducible AI research. EUCAIM complements these existing resources since it provides a hybrid federated–centralised infrastructure with common standards for anonymisation, metadata, clinical linkage and data quality, enabling continuous expansion of the atlas and supporting cross-border and GDPR compliant analysis at scale.”

**Reviewer 3.2**: Describe more in detail the system for hosting and using the data. The authors distinguish between two options (materials and method, paragraph 1): 1) DSA, where the data stays in the system, or DTA, where the data goes to the federated node. Can you provide more technical information about this part? Or reference to already published protocols/papers describing this part? Some questions here: Why these two options and not only DTA directly? For hospitals choosing 1), is it up to them to buy a server to host the data? How is the connection then to other hospitals willing to use the data through the DSA? Does EUCAIM provide a technical team to make all these installations in the local computers, or the hospital should do it? How can other hospitals use the DSA data to train models if the data did not leave the hospital? For DTA data, can I download the data into my servers to perform research (e.g. trained AI models)?

 I understand that the paper here focuses on the population of the atlas, but some clarification (a paragraph will be enough) here is important, so that the community understands how the data can be used later. Otherwise, it seems a very nice platform to store data, but the article does not give any clue about the potential to use the data later **.**

**Authors:** To address this comment, we have included the following paragraph on page 6: “The choice between a DSA and a DTA reflects the heterogeneous technical capabilities and data governance preferences of participating hospitals. Some institutions require that data processing remains fully under their control; for these cases, EUCAIM enables the deployment of a federated node within the hospital infrastructure, hosted on local servers and integrated with the EUCAIM platform through secure, encrypted channels. This environment operates as a Secure Processing Environment under the EHDS framework, allowing authorised users to run analytics and train models locally without any image data leaving the institution. EUCAIM provides technical specifications, containerised services, and remote support to assist hospitals in the installation and configuration of these nodes, although the hardware is typically procured and maintained by the institution according to its internal policies. Other hospitals opt for a DTA, transferring data to the EUCAIM reference node where harmonisation, storage and computation are centrally managed. While software models trained within this environment may be exported subject to the Access Committee approval, original imaging data are never downloadable to external servers. This dual approach offers flexibility for centres with different resources and legal constraints while ensuring that all data, whether local or centralised, can be incorporated into cross-site analyses through a unified and privacy-preserving architecture.”

**Reviewer 3.3: **Disclose or add reference for already available material. This is published in Open Research journal, so I guess that the goal is to make as open as possible the results presented here. There are many parts where you mention that the authors (or the EUCAIM team) have developed a lot of material, but no reference is given. For instance, in material and methods “For data standardization, all DICOM files and metadata were validated for compliance with EUCAIM structure”, “The DICOM File Integrity Checker developed by our group” → is this material published elsewhere or made open-source through a github/github or similar repository? The amount of work that has been developed here has an incredible value for the community and should be made public whenever possible. 

**Authors: **Thank you for pointing this out. Additional references have been incorporated on page 6 to clarify the availability of the tools and materials mentioned.

-
  - DICOM File Integrity Checker: The DICOM File Integrity Checker developed by the group is not a public tool; however, it is accessible to EUCAIM users for data preprocessing within the project. The manuscript now includes a reference to its entry in the EUCAIM bio.tools catalogue:

 
https://bio.tools/dicom_file_integrity_checker_by_gibi230

-
  - EUCAIM Handbook: A reference to the EUCAIM Handbook has been added. This resource provides a detailed description of the data standardization workflow and the tools available within the project: https://eucaim.gitbook.io/handbook

**Reviewer 3.4: **Pseudonymisation versus Anonymisation. I have a question regarding this, since both are mentioned during the text, it is not clear to me if the data uploaded is pseudonymised, and there is a log file somewhere (in the hospital submitting the data) that keeps a link (e.g. PatientID) between the data submitted and the real patient identity, or the data is fully anonymised. If the data is totally anonymised and there is no log-file, how do we prevent errors in the future if the hospital submit the same patient (e.g. submission in 2025 for project X, new patient ID projectX_01 - submission in 2026 of same patient for project Y, new patient ID project Y_01). Also, besides removing DICOM tags, can you describe if any action on the images has been done or not? There are some people that advocate to remove facial information from head-and-neck scans, but this can affect the potential use of the data (e.g. in head-and-neck cancer patients).**** **Authors: **To address this comment, we have included the following explanation and sentences on The Data extraction and exposure pipeline (page 6): “The data preparation workflow includes a two-stage privacy-preserving process:

1. Local pseudonymisation:

Pseudonymisation is performed on a restricted-access Virtual Machine by authorized personnel of the Experimental Radiology and Imaging Biomarkers Platform (PREBI) using an in-house tool developed by the Biomedical Imaging Research Group (GIBI230). The process includes: replacement of PatientID, PatientName and AccessionNumber with specific pseudonym and hashes using Blake2b, renaming of folder structures to remove personally identifiable information (PII), removal of public and private DICOM metadata that may contain PII, exclusion of screenshots (ImageType = SCREEN SAVE) and manual review of secondary captures (DERIVED or SECONDARY). This step ensures consistent pseudonymised identifiers for repeated submissions of the same patient at the hospital level. The mapping between original and pseudonymised identifiers is maintained only temporarily by the hospital IT service and is not accessible externally.

1. EUCAIM anonymization:

Once pseudonymised data are transferred to the Pseudonymised Medical Imaging Repository, the EUCAIM anonymization pipeline is applied. This pipeline enforces the EUCAIM DICOM Anonymization Profile and generates a unique hash per patient, linked to the project and site, with no retained traceability. Sensitive DICOM headers are removed, and OCR-based pixel-level text detection can be applied to eliminate “burned-in” personal information. File integrity checks and pixel-level duplicate detection can be performed to prevent repeated inclusion of the same images across different projects.” We have also reorganized this section adding the following sentence: “This two-layer anonymization approach mitigates re-identification risks, aligning with procedures recommended by the Spanish Data Protection Agency and best practices from the Singaporean authority ( Figure 1).  

*https://openreseurope-files.f1000.com/linked/263811.image_1.gif*

**Figure 1. Steps for anonymization.** Source: Adapted from AEPD. Guide to basic anonymization. Prepared by the National Data Protection Authority of Singapore (PDPC - Personal Data Protection Commission Singapore. Additionally, after transferring it to Pseudonymised Medical Imaging Repository and anonymising the datasets, the DICOM File Integrity Checker developed by our group was used to detect corrupted or missing files. Data were finally ingested into EUCAIM by transferring them to the Reference Node through the QP-Insights API, or by sharing them through a federated node.. For data standardization, all DICOM files and metadata were validated for compliance with EUCAIM’s structure and interoperability standards. The data preparation process and the tools involved are defined in the EUCAIM Handbook. Additionally, the metadata of all datasets were registered in the EUCAIM Public Catalogue, which follows the Health DCAT-AP standard and ensures compliance with FAIR principles at the dataset level. An additional layer of dataset discoverability is provided to EUCAIM Data Users through the Federated Query tool, which enables them to perform queries based on specific criteria and retrieve the number of cases (as aggregated numerical results) that meet those criteria”

**Reviewer 3.5: **Data quality and documentation. You do mention DICOM compliance, anonymisation, etc, but what about data quality and documentation?  About data quality, is there any metadata or any quality check to measure the quality of the submitted data? I am thinking more on the annotations of the data, for instance, if the annotations have been done by experts and following consensus international guidelines, etc A concrete example would be the adherence to contouring guidelines in radiotherapy images. Data annotations not being compliant to these guidelines and used to train models might carry future problems. Also, we could think about data quality of the images, if a hospital submit images with very low resolution, artefacts, etc. A way to go would be to enable a community rating for each dataset … About data documentation. I am putting myself on the user side, and the first question that comes to my mind is: how do I find and decide which data I use for my project? Having data documentation is very important, like a Data Sheet (similar to what they have done in https://arxiv.org/abs/1803.09010, https://datanutrition.org/, …). Is there any similar documentation standard in EUCAIM?

**Authors: **We have reviewed your relevant comment about data quality and we would like to add that efforts are ongoing to define and implement European-level guidelines for data quality, such as those being developed within the QUANTUM project. At the time of dataset preprocessing for this manuscript, these guidelines were not yet applied. In addition, EUCAIM Data Users have access to other tools and workflows to assess data quality. We have included the following comment on the manuscript on page 8: “This rigorous ETL pipeline ensures scalability, data quality through the access to tools and workflows within EUCAIM” In relation with data augmentation, we would like to confirm that EUCAIM provides dataset metadata in the Public Catalogue, which follows the Health DCAT-AP standard and FAIR principles. Researchers can explore datasets and evaluate suitability using the Federated Query tool, which returns aggregated counts for specific criteria, supporting informed dataset selection. We have included the following comment on the manuscript on page 6:” The data preparation process and the tools involved are defined in the EUCAIM Handbook. Additionally, the metadata of all datasets were registered in the EUCAIM Public Catalogue, which follows the Health DCAT-AP standard and ensures compliance with FAIR principles at the dataset level. An additional layer of dataset discoverability is provided to EUCAIM Data Users through the Federated Query tool, which enables them to perform queries based on specific criteria and retrieve the number of cases (as aggregated numerical results) that meet those criteria”

# Reviewer report: Reviewer response for version 1

**DOI:** 10.21956/openreseurope.22734.r63504

**Published:** 2025-11-11

**Authors:** Michal Strzelecki

**Affiliations:** 1 Lodz University of Technology, Lodz, Poland

The paper addresses a highly significant issue concerning access to medical imaging data. Such data are essential for the development and implementation of AI algorithms that support diagnostic imaging. Through the EUCAIM project, an effective method for accessing these data has been devised, ensuring compliance with extremely stringent legal and ethical requirements while overcoming technical challenges—primarily related to incompatibilities among hospital PACS systems.

 The case study described demonstrates that access to such data is indeed feasible, although at this stage it encompasses only approximately 12,000 images. However, the authors’ conclusions are not optimistic. They anticipate difficulties in scaling the developed data-sharing process for broader clinical implementation, due to diverse data management models within medical institutions and legislative discrepancies governing access to medical data.

 As a result, narrowing the gap in the development of such algorithms between Europe and North America or certain Asian countries may become increasingly challenging. Nevertheless, the reviewed work is of considerable importance for the advancement of diagnostic imaging, and its indexing is strongly recommend.

Is the case presented with sufficient detail to be useful for teaching or other practitioners?

Yes

Is the work clearly and accurately presented and does it cite the current literature?

Yes

If applicable, is the statistical analysis and its interpretation appropriate?

Not applicable

Are all the source data underlying the results available to ensure full reproducibility?

Yes

Are the conclusions drawn adequately supported by the results?

Yes

Is the background of the case’s history and progression described in sufficient detail?

Yes

Reviewer Expertise:

medical imaging, AI, machine learning

I confirm that I have read this submission and believe that I have an appropriate level of expertise to confirm that it is of an acceptable scientific standard.

# Reviewer report: Reviewer response for version 1

**DOI:** 10.21956/openreseurope.22734.r63501

**Published:** 2025-11-11

**Authors:** Neha Neha, Deepak Kumar Shukla

**Affiliations:** 1 Rutgers Business School, Rutgers University Newark Business School (Ringgold ID: 33869), Newark, Jersey, USA; 2 Kent State University, Kent, USA

This article documents the implementation of the European Federation for Cancer Images (EUCAIM) initiative, focusing on the integration of imaging and clinical data from a reference university hospital into the EUCAIM infrastructure. 

 Good points are:

 1. High data-processing throughput (12,484 studies, 98.6% efficiency).

 2. Clarity and structure suitable for replication within similar EU projects.

 3. Integrates lessons from multiple large-scale initiatives.

 However Recommendations for Revision:

 There is minimal discussion on computational performance metrics or storage requirements. Also;

 1. provide pseudonymization scripts, metadata dictionaries, or anonymized examples through Zenodo or EUCAIM’s public repositories.

 2. include workflow diagrams or tabular summaries of ethical and legal documentation steps.

 3. address sustainability models and future interoperability challenges beyond current pilot hospitals.

 3. ensure consistent acronym expansion.

Is the case presented with sufficient detail to be useful for teaching or other practitioners?

Partly

Is the work clearly and accurately presented and does it cite the current literature?

Yes

If applicable, is the statistical analysis and its interpretation appropriate?

Not applicable

Are all the source data underlying the results available to ensure full reproducibility?

Partly

Are the conclusions drawn adequately supported by the results?

Yes

Is the background of the case’s history and progression described in sufficient detail?

Yes

Reviewer Expertise:

Artificial IntelligenceComputer VisionImage ProcessingDeep learningMedical Imaging

We confirm that we have read this submission and believe that we have an appropriate level of expertise to confirm that it is of an acceptable scientific standard, however we have significant reservations, as outlined above.

## References

[1] : Retrieval-Augmented Generation (RAG) in Healthcare: A Comprehensive Review. *AI* .2025;6(9) : 10.3390/ai6090226 10.3390/ai6090226
